# Stimulatory Effects of Oleci Acid and Fungal Elicitor on Betulinic Acid Production by Submerged Cultivation of Medicinal Mushroom *Inonotus obliquus*

**DOI:** 10.3390/jof7040266

**Published:** 2021-03-31

**Authors:** Hanghang Lou, Hao Li, Tianyu Wei, Qihe Chen

**Affiliations:** Department of Food Science and Nutrition, Zhejiang University, Hangzhou 310058, China; louhanghang@zju.edu.cn (H.L.); 21513047@zju.edu.cn (H.L.); 21913067@zju.edu.cn (T.W.)

**Keywords:** medicinal mushroom, *Inonotus obliquus*, betulinic acid, oleic acid, fungal elicitor

## Abstract

To evaluate the novel strategy of oleic acid and fungal elicitor (made from *Aspergillus niger*) to elicit betulinic acid biosynthesis in medicinal mushroom *Inonotus obliquus*, we conduct the stimulatory effects investigation for synthesizing betulinic acid from betulin. HPLC results indicated oleic acid and fungal elicitor were effective stimulators. The supplementation of 1.0 g/L oleic acid led to the highest increase of betulinic acid either in dry mycelia or fermentation broth by 2-fold of the control. Fungal elicitor at 45 mg/L markedly increases mycelia growth by 146.0% and enhance intracellular betulinic acid accumulation by 429.5% as compared to the controls. Quantification of transcription levels determined that oleic acid, fungal elicitor and their combinations could induce the expressions of key genes involved in betulinic acid biosynthesis, such as HMG-CoA reductase and squalene synthase. These findings indicated that oleic acid and fungal elicitor could enhance betulinic acid metabolism by up-regulating key genes expression.

## 1. Introduction

*Inonotus obliquus*, a commonly known as chaga medicinal mushroom, without any known side effects, is a white-rot fungus belonging to the family *Hymenochaetaceae, Donk*. As a kind of functional food and traditional Chinese herb, *I. obliquus* has been utilized for a long time [1]. This medicinal mushroom is widely distributed in Asia, North Europe and North America at latitudes of 45–50 °N. Russians have used it as a folk therapy since the sixteenth century for the treatment of cancer, diabetes and heart diseases [2,3]. Recently, it has been reported that *I. obliquus* contained above ten terpenoids, which included betulinc acid (BA) [4]. Due to its slow growth and rarity in nature, *I. obliquus* is not a reliable source of industrialized production of betulinic acid. Liquid culture of this higher fungus can be a potential substitute for the efficient extraction of BA. BA is a secondary metabolite which is greatly influenced by environmental conditions, such as sunlight, low temperature and stimulation of other microbes [5]. Therefore, novel methods of the submerged fermentation of *I. obliquus* are vital to improve BA production.

Recently, it has attracted great attention to combining inducing factors in the submerged culture of *I. obliquus* [5,6,7,8]. Fatty acids have been proved to accelerate mycelia growth of several medicinal fungi such as *G. lucidum* [9]. Moreover, fatty acids were found to improve the production of some metabolites which include triterpenoids [7,10]. Oleic acid could not only accelerate the growth of *I. obliquus*, but also improve the production of terpenoids [7]. Betulin is the precursor material of BA. In recent times, several reports have demonstrated that BA could be prepared through biotransformation process [11,12,13]. Meanwhile, betulin was used stimulatory agent to enhance mycelial biomass and production of exo-polysaccharides from *I. obliquus* [8]. Another potential stimulator may be fungal elicitor, which has been applied in *G. lucidum* [14]. Some reports suggested that fungal elicitor had an influence on both mycelia growth and secondary metabolites accumulation [15,16,17]. Up to now, several genes related to the biosynthesis of BA have been reported, such as farnesyl pyrophosphate synthase (FPS), HMG-CoA reductase (HMGR) and squalene synthase (SQS) [7,18,19]. Nevertheless, there are few studies focusing on investigating the effects of these compounds on BA biosynthesis and the inducing mechanism of it in *I. obliquus* [7].

BA is a kind of pentacyclictriterpenoid compound, which has drawn increasing attention due to its various bioactivities, such as anti-inflammatory, anti-HIV [20], anti-cancer, anti-melanoma [21], antimicrobial, antihelmintic [22] and antineoplastic [23,24] activities, and the most importantly does minimal harm to normal cells [25]. Interestingly, BA is able to prevent fat accumulation by means of fat modulation and carbohydrate metabolism [26,27]. Accordingly, BA is regarded as a leading compound against HIV infection and a wide range of tumor cells for further preclinical and clinical studies [28]. Presently, a large number of studies have demonstrated that BA could induce cell apoptosis, such as microglia BV-2 cells [29], human prostate cancer cells [30], PC12 cells [31], human leukemia HL-60 cells [32], human colon carcinoma cells [33] and so on. Previous studies have also indicated that the alternation of mitochondrial membrane potential (MMP) has a relationship with cell apoptosis [34,35]. Moreover, BA induces cell apoptosis mainly through a mitochondrial pathway, such as PC12 cells [31] and MCF-7 cells [36]. 

In this work, with the purpose of enhancing mycelia accumulation and the production of BA, a variety of stimulating methods were attempted in the liquid fermentation of *I. obliquus*. Fungal elicitor (made from *Aspergillus niger*), oleic acid and betulin were supplemented during the submerged culture of *I. obliquus*. HPLC-MS was used for the confirmation of the existence of BA in *I. obliquus*. This work also explores the optimal approach to increase mycelia growth and BA formation, as well as the possible biosynthesis mechanism of BA in *I. obliquus*.

## 2. Materials and Methods

### 2.1. Chemicals and Reagents

Standard BA (≥97%) was procured from Tokyo Chemical Industry Co. Ltd., (Tokyo, Japan). Standard betulin (≥98%) was bought from Sigma-Aldrich Corporation (St. Louis, MO, USA). Methanol and acetonitrile were both obtained from Sanli Chemical Co. Ltd., (Zhejiang, China). Dimethyl sulphoxide (DMSO), acetonitrile and methanol were all chromatographically pure. Apart from these, other chemicals used in this study were of analytical grade. 

### 2.2. Microorganism and Culture Media

*I. obliquus* CFCC 83414 is purchased from China Forestry Culture Collection Center. The agar slants used for the activation of *I. obliquus* were a mixture of potato dextrose agar (PDA) and the aqueous extracts of bran. The percentage of raw material composition of solid culture medium was PDA (1% potato extract powder, 2% glucose, 1.8% agar, 0.01% chloramphenicol) and the aqueous extracts of wheat bran, which was prepared as follows: wheat bran was added into pure water with a ratio of 5%, then boiled and filtered. After that, the aqueous extracts of bran were combined into PDA. The liquid medium used for the growing of *I. obliquus* was a mixture of potato dextrose broth (PDB) medium and the aqueous extracts of wheat bran. The liquid medium consists of 0.8% potato extract powder, 2% glucose, and the rest was the aqueous extract of wheat bran. Firstly, wheat bran was added into pure water with a ratio of 5%, then boiled and filtered it, and removed impurities. After PDB was supplemented into the aqueous extracts of wheat bran,100 mL was inoculated into 500 mL conical flasks. All fermenation media should be sterilized at 121 °C for 20 min before use.

### 2.3. Microorganism and Culture Media

*I. obliquus* was cultivated on the agar slants at 25 °C in the dark for two weeks. The obtained slants were stored at 4 °C and activated every two months. For the liquid culture, *I. obliquus* was incubated in 500 mL flasks with 100 mL fermentation media at 25 °C in the dark for 8–14 days. As for rotating speed, the initial 2 to 3 days was 0 rpm, then 100 rpm for 3 to 4 days and the left days 180 rpm. To assure the stability, each inoculated fungi should be derived from the same batch.

### 2.4. Preparation of Standard Solutions and Elicitors

Betulin was dissolved in DMSO in the concentration of 5 mg/L, which was sterilized by 0.22 μm Sterile Millipore filter and added 2 mL/L to the culture media on day 6. Betulin solution was added to the culture media and the final concentrations of betulin were 10, 25 and 40 mg/L respectively. Oleic acid was added at the concentration of 1.0, 2.0 and 3.0 g/L.

*A. niger* was inoculated on PDA slant medium and cultivated at 25 °C for 7 days, then transferred to PDB liquid medium and cultured at the shaking speed of 140 rpm at 25 °C for 7 d. The mycelia were washed three times with distilled water, and disrupted with a JY96-IIV ultrasonic cell pulverizer (300 W, 1 min × 5 times). The precipitation was obtained by centrifuging at 12,000× *g* for 10 min. Under the microscope, the culturing mycelia should be fragmented, and then washed three times with the distilled water. The cleaned mycelia were supplemented to distilled water, which was sterilized at 121 °C for 20 min. Fungal elicitor was acquired and reserved at 4 °C with the concentration of 10 mg/mL. BA was dissolved with methanol at the concentration of 5.0 mg/mL, which was kept at 4 °C in darkness as a stock solution.

### 2.5. Assay of Mycelia Biomass and the Isolation of BA

As biotransformation process ended, the fermentation broth and the cultured mycelia were separated with centrifuge at 12,000× *g* for 10 min. Then mycelia were washed with distilled water for three times and dehydrated to constant weights by freezing dryer for 2 d. The dry weight of mycelia should be measured. Afterwards, the dry mycelia were mixed with some distilled water and smashed by ultrasonic cell pulverizer (300 W, 1 min × 5 times). The fermentation broth and the mycelium broken liquid were mixed with equal amounts of ethyl acetate respectively and extracted twice by ultrasound at 25 °C for 30 min each time. All the organic phase was merged and concentrated by rotary evaporation under vacuum at 55 °C. At last, the obtained remains were dissolved by 1.5 mL methanol and filtrated with a 0.22 μm millipore filter, then analyzed by RP-HPLC and identified by HPLC-MS. 

### 2.6. Reverse-Phase HPLC (RP-HPLC) and HPLC-MS Analysis of BA

BA was detected by RP-HPLC referred to the literature [37]. The RP-HPLC instrument mentioned in this experiment is made up of two Waters 510 pumps (Waters, Milford, MA, USA), a sample injector (Rheodyne, Cotati, CA, USA) with a 20 μL loop, and a Waters 996 photodiode array detector. The chromatographic column applied in this study is a reversed phase Symmetry C18 (4.6 mm × 250 mm, 4 μm, Waters). The mobile phase, acetonitrile and water at a percentage of 91 to 9 (*v*/*v*), is filtrated by means of a 0.22 μm millipore filter, degassed by ultrasonication. The normal operating pressure is between 90 and 120 MPa. The velocity of flow is 1.0 mL/min, below 30 °C generally. For each sample, the injection volume is 10 μL and the running time is 25 min. The detection wavelength was 210 nm. The chromatographic peaks of BA were determined by contrasting the retention time with the known standards of BA. The concentration of BA in each sample was calculated by the standard curve line of BA, which was set up on the basis of peak areas. For HPLC-MS, the part of HPLC was similar to that of RP-HPLC. For Mass Spectrum (MS), negative ion electrospray ionization (ESI) was selected to form deprotonated molecules [M-H]^—^ at m/z 455.35 (BA) [38].

### 2.7. Real-Time Fluorescence Quantitative PCR Analysis

30–100 mg of filamentous fungi that grew in the submerged media which contained oleic acid at the concentration of 1.0 g/L, fungal elicitor with the addition of 45 mg/L and the combination of them were sampled respectively. Total RNA of the fungi was extracted with Axyprep multisource total RNA miniprep kit (Corning, Corning, NY, USA). The purity of RNA was analyzed with SIM-100 Bioanalyzer (Hangzhou SIMGEN Biotech Co., Ltd., Hangzhou, China). First-strand cDNA was synthesized with 500 ng RNA with the kit of HiScript^®^ Q RT SuperMix for qPCR (Vazyme Biotech Co., Ltd.). RT-qPCR was carried out by applying AceQ^®^ qPCR kit, whose dyestuff was SYBR Green I (Vazyme Biotech Co., Ltd., Nanjing, China). In addition, primers were designed according to the software of Primer 6. The sequences of forward and reverse primers were as follows: β-actin-F‘CCAGCCATCGTTCCTTGGACTT’, β-actin-R‘TCGTACCACCAGACAGCACAAC’;HMGR-F‘ACATCCTCACGGCGATCTTCCT’,HMGR-R‘GCGTCATCATTCGTTGGCTCCA’;SQS-F‘AGCAGGTGTGACGGCAAACG’,SQS-R‘GACGATGGCGAACGCAAGGA’.

The accession numbers of β-actin, HMGR and SQS are KP970553.1, JN580310.1 and KC182754.1, respectively. The whole process of RT-qPCR consists of three stages: a pre-degeneration step at 95 °C for 3 min, 40 cycles at 95 °C for 10 s, 60 °C for 30 s, and one cycle at 95 °C for 15 s, 60 °C for 60 s and 95 °C for 15 s. The relative expression of mRNA was analyzed according to the 2^−ΔΔCt^ method put forward by Livak and Schmittgen [39].

### 2.8. Statistical Analysis

All experiments were conducted at least triplicate to increase the credibility of results. Each point is the mean of duplicate experiments.

## 3. Results

### 3.1. Comparison of Different Exogenous Inducing Factors

**Figure 1:**
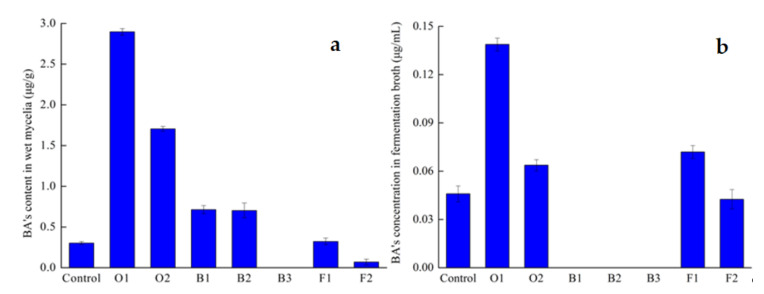
Effects of different exogenous inducing factors on the induction of BA’s content in wet mycelia (**a**) and fermentation broth (**b**). O1, oleic acid 1.0 g/L; O2, oleic acid 2.0 g/L; B1, betulin 10 mg/L; B2, betulin 25 mg/L; B3, betulin 40 mg/L; F1, fungal elicitor 45 mg/L; F2, fungal elicitor 90 mg/L.

To explore the effects of exogenous factors on BA production, various concentrations of oleic acid (1.0, 2.0 g/L), betulin (10, 25, 40 mg/L) and fungal elicitor (45, 90 mg/L) were supplemented to the liquid culture media on 6th day, respectively. The data presented in Figure 1 on the yield of BA were determined on 13th day. As shown in Figure 1, there was an obvious difference in BA biosynthesis between the control and the different experimental groups with seven treatments. In contrast to the control, oleic acid at the concentration of 1.0 and 2.0 g/L could increase the content of BA in wet mycelia by 8.57-, 5.63-fold and the concentration of BA in fermentation broth by 3.02-, 0.39-fold individually. At the concentration of 10 and 25 mg/L, betulin increased BA productions in wet mycelia by 135.7% and 132.2% respectively. While the concentration of betulin was 40 mg/L, BA was detected neither in mycelia nor in fermentation broth. The fungal elicitor concentration of 45 mg/L increased the production of BA either in wet mycelia or the fermentation broth, resulting in 6.7% and 56.9% higher than the untreated control group. The addition of fungal elicitor at 90 mg/L showed an inhibitory effect on BA production. 

Among the three kinds of stimulators investigated, oleic acid and fungal elicitor were more effective to improve BA formation in both mycelia and fermentation broth than betulin. As a consequence, oleic acid and fungal elicitor were selected for further investigation.

### 3.2. The Identification of BA from I. obliquus

Due to the interference of similar peaks in the HPLC chromatograms, the identification of BA was essential. HPLC-MS was reported to be applied to identify BA [40]. Therefore, HPLC-MS method was used to confirm the existence of BA in this work. A representative sample and an internal standard were detected in this study. 

**Figure 2:**
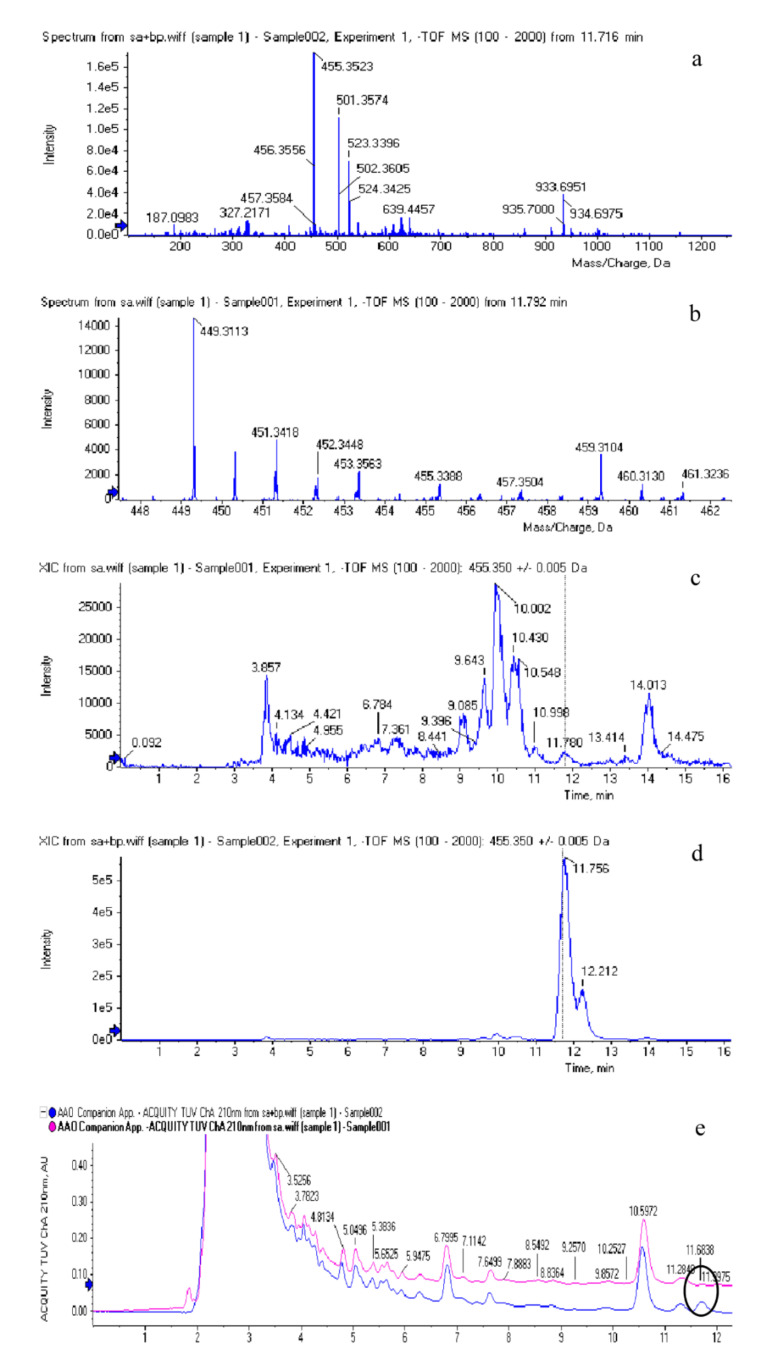
Deprotonated molecular ions in the mass spectra for BA in (**a**) internal standard (IS) and (**b**) sample, Chromatograms of BA in sample (**c**) and IS (**d**). Peaks of BA in sample and IS in the same chromatogram (**e**).

At this stage, owning to the stability and accuracy of mass spectra, negative ion electrospray ionization (ESI) was selected to form deprotonated molecules [M-H]-at m/z 455.35 both in sample and internal standard (IS), as presented in Figure 2a,b respectively. At 11.7 min nearby, deprotonated molecules [M-H]-of m/z 455.35 were detected both in sample and IS, which certified the existence of BA because BA’s ratio of mass to charge was 455.35. In turn, Figure 2c,d confirmed the existence of BA’s chromatographic peaks when the ratio of mass to charge was 455.35. Figure 2e showed the identity of BA from another perspective. Moreover, at 11.7 min nearby, there were separated peaks, where the upper one (sample) was lower than the beneath one (IS). In summary, Figure 2 further confirmed the existence of BA in *I. obliquus*.

### 3.3. Determination of Optimal Adding Concentration of Oleic Acid, Fungal Elicitors

**Figure 3:**
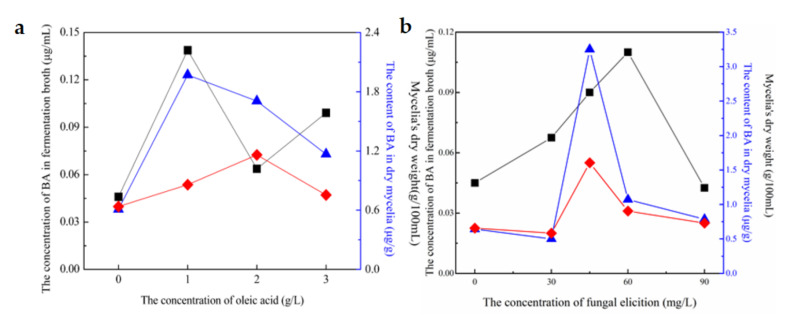
Effects of oleic acid’s concentration (**a**) and fungal elicitor’s concentration (**b**) on fermentation progress. 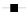 BA’s concentration in fermentation broth; 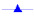 BA’s content in dry mycelia; 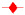 Mycelia’s dry weight.

Figure 3 presented the effects of the different concentrations of oleic acid (Figure 3a) and fungal elicitor (Figure 3b) on mycelia growth and BA production both in dry mycelia and fermentation broth. Oleic acid and fungal elicitor were supplemented to the culture media on the 6th day of full fermentation period. Figure 3a revealed that oleic acid had a positive effect on BA accumulation and mycelia biomass at all the investigated levels (1.0–3.0 g/L). Though 2.0 g/L oleic acid caused the most biomass (182.3% of the control), 1.0 g/L oleic acid could bring the highest increases of BA content in dry mycelia and fermentation broth by 223.1% and 202.0% compared to the control separately. As a whole, the optimal concentration of oleic acid can be 1.0 g/L. Figure 3b represented the influence of the concentrations of fungal elicitor on BA biosynthesis and mycelia biomass. 45 mg/L fungal elicitor resulted in an increase in mycelia biomass (428.9%) and BA concentration (146.0%) in dry mycelia compared to the controls. The addition of 60 mg/L fungal elicitor could bring the highest increase of BA biosynthesis in fermentation broth by 143.7%. When the concentration arrived at 90 mg/L, fungal elicitor slightly increased biomass and BA production. In general, the optimal concentration of fungal elicitor can be 45 mg/L. This finding also confirmed that the best condition for mycelia growth could not be necessarily beneficial for BA biosynthesis. The effects of oleic acid and fungal elicitor on mycelia growth and BA accumulation were dose-dependent.

### 3.4. Effect of Addition Time

**Figure 4:**
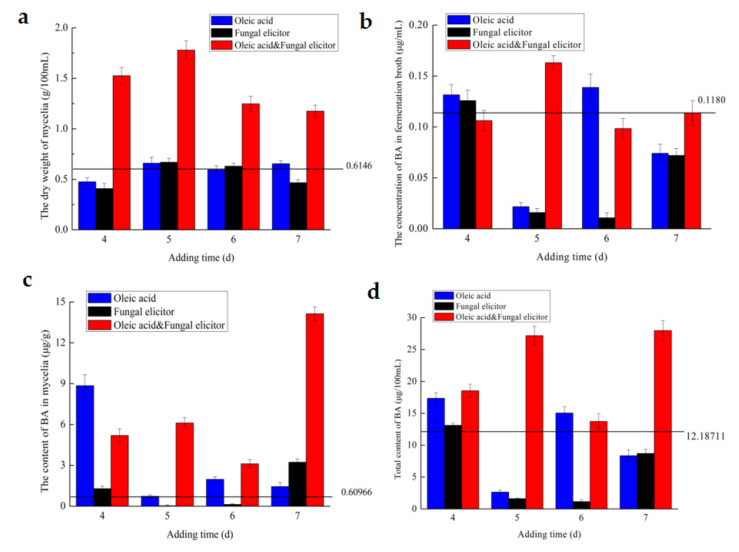
Effect of inducer’s adding time on mycelia’s dry weight (**a**), BA’s content in dry mycelia (**b**), BA’s concentration in fermentation broth (**c**) and BA’s total content (**d**).

Apart from addition amounts, supplementation time had a great influence on mycelia growth and BA production. To find the best addition time of the inducers, oleic acid of 1.0 g/L, fungal elicitor of 45 mg/L and the combination of these two stimuli were supplemented into the liquid culture medium on the 4th, 5th, 6th, 7th day respectively. Figure 4 showed the influences of addition time of inducers on mycelia growth, BA’ s accumulation in mycelia, BA’s concentration in fermentation broth and total BA’s contents, respectively. 

In Figure 4a, the complement of oleic acid increased mycelia biomass by 7.2% when it was added on 5th day. Fungal elicitor resulted in an increase in mycelial dry weight by 8.8% compared to the control when it was supplemented on 5th day. The combination of these two stimuli could increase the growth of *I. obliquus* most potently by 189.4% when they were replenished on 5th day. Figure 2b showed that the addition of oleic acid on 4th day could markedly increase BA accumulation by 13.5-fold compared to the control. Fungal elicitor could increase BA level by 4-fold compared to the control when it was complemented on 7th day. As *I. obliquus* was treated with the combination of these two stimuli on 7th day, BA production in mycelia reached to the highest level, which was 22.2-fold higher than the control. In Figure 4c, if oleic acid was replenished on 6th day, there would be an increase of 17.6% in BA production in fermentation broth compared with the control. Fungal elicitor could increase BA production in liquid media by 6.6% only when it was added on the 4th day. The highest production of BA in liquid culture media was achieved when the two stimuli combination was conducted on 5th day, and was 1.38 times higher than the control group. Oleic acid was able to increase the total content of BA by 41.1% and 23.5% when added on the 4th and 6th day (Figure 4d). The integrated two stimulators gave increases in BA total content distinctly by 129.7% of the control when they were complemented on 7th day, respectively. Conclusively, the best addition time of oleic acid, fungal elicitor and the combination of oleic acid and fungal elicitor is 4th, 4th and 7th day, respectively.

### 3.5. Effect of Culture Age

**Figure 5:**
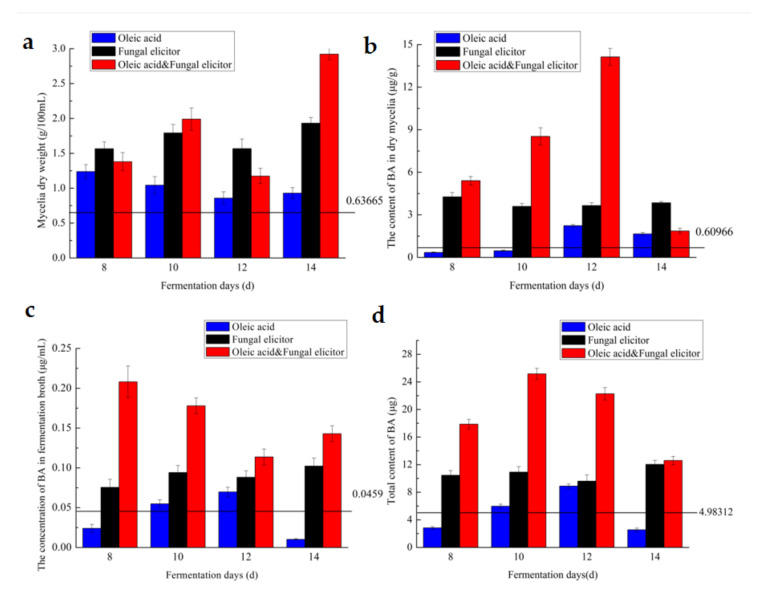
Effects of fermentation time on mycelia’s dry weight (**a**), the content of BA in dry mycelia (**b**), of the concentration of BA in fermentation broth (**c**) and the total content of BA (**d**).

To determine the fermentation period, oleic acid of 1.0 g/L, fungal elicitor of 45 mg/L and the combination of these two inducers were added on the 6th day and the fermentation process ended on the 8th, 10th, 12th and 14th, respectively. Figure 5 displayed the effect of fermentation period on mycelia growth of the *I. obliquus* and BA accumulation. Figure 5a–d displayed the effects of fermentation period on growth of the *I. obliquus*, BA’s content in mycelia/fermentation broth and BA’s total content, respectively.

Data presented in Figure 5a, it was found that the addition of oleic acid could increase biomass potently by 94.5% on 8th day if compared to the control. Fungal elicitor could lead to the highest increase in mycelia dry weight by 203.6% of the control on 14th day. The combination of these two stimuli could boost the growth of *I. obliquus* most effectively on 14th day, with an increase of 359.0%. Oleic acid brought an increase of BA in mycelia by 2.7- and 1.7-fold of the control on 12th and 14th day, respectively (Figure 5b). Fungal elicitor could bring the highest BA production in mycelia biomass with an increase of 6.0-fold on 8th day. The highest BA production in mycelia was obtained on 12th day with the addition of two stimulators combination and was 21.2-fold higher than the control. In Figure 5c, for BA’s concentration in fermentation broth, oleic acid, fungal elicitor and the combined two stimuli could lead to the highest increases on 12th, 14th and 8th day, which were 51.9%,122.9% and 353.4% higher than the control, respectively. Moreover, oleic acid, fungal elicitor and the combination of them extremely increased the total BA levels by 78.6%, 141.6% and 404.9%, respectively (Figure 5d). On the whole, the optimized fermentation time greatly increased the BA level in each sample as compared to that in the control group. The optimal fermentation periods are 12, 14 and 10 days with the additions of 1.0 g/L oleic acid, 45 mg/L fungal elicitor and the combination of them individually.

### 3.6. Transcriptional Responses to Different Stimulators

**Figure 6:**
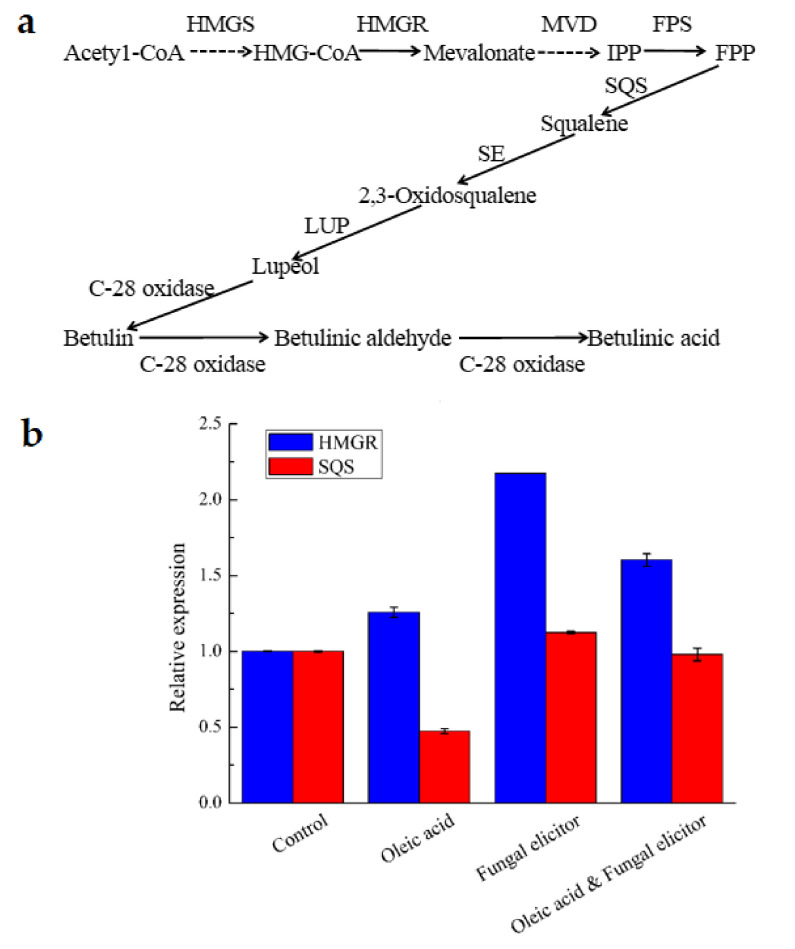
(**a**) BA biosynthetic pathway in *I. obliquus*. Dashed lines represent steps made up of several enzyme reactions. HMG-CoA, hydroxy-3-methylglutaryl-Coenzyme A; HMGS, HMG-CoA synthase; HMGR, HMG-CoA reductase; MVD, mevalonate-5-pyrophosphate decarboxylase; IPP, isopentenyl-pyrophosphate; FPS, farnesyl pyrophosphate synthase; FPP, farnesyl diphosphate; SE, squalene epoxidase; SQS, squalene synthase; OSC, oxidosqualene cyclase; LUP, lupeol synthase. (**b**) Quantitative real-time PCR analysis of genes involved in the biosynthesis pathway of BA.

Most reports related to fungi-derived BA focus on BA extraction and its bioactivities. Accordingly, the underlying mechanism of BA biosynthetic pathway in fungi, especially in *I. obliquus*, is poorly understood. As a type of triterpenoid, BA is synthesized via the mevalonate pathway (Figure 6a). The anterior study reports that the genes encoding the key enzymes, such as HMGR and SQS, involved in BA biosynthesis were successfully cloned from *I. obliquus* [18]. In this work, the transcription levels of these genes were investigated under a variety of situations including 1.0 g/L oleic acid, 45 mg/L fungal elicitor and the integration of them. Figure 6b shows that all of three treatments enhanced the expressions of HMGR gene. However, the expression of SQS gene was up-regulated only by fungal elicitor induction. Moreover, the highest transcriptional levels for HMGR and SQS were observed at 45 mg/L fungal elicitor, where levels were 2.17- and 1.13-fold of the control respectively. In addition, the transcriptional level for HMGR at the combination of oleic acid and fungal elicitor was 1.6-fold compared to the control, which was in the middle among three treatments. In contrast, the expression level of SQS gene was almost the same as that of the control. To sum up, the supplementation of 1.0 g/L oleic acid, 45 mg/L fungal elicitor and the combination of them could increase the expression level of HMGR gene by 25.7%, 117.3%, and 60.4%, respectively. The fungal elicitor at 45 mg/L could also enhance the transcriptional levels of SQS by 12.5%.

## 4. Discussion

In the previous study, several triterpenoids were isolated from the extract of *I. obliquus* and one of them was identified to be BA [41]. With the application of HPLC-MS, BA was certified to be one of the most important products of *I. obliquus* once again. Of course, apart from BA, there are other isolated peaks worthy of being identified, which needs further study. For higher fungi, it was reported that BA production could be improved from betulin by biotransformation in cultured *Armillaria luteo-virens* Sacc cells [42], *Aspergillus foetidus*, *Aspergillus oryzae* [11] and *Cunninghamella blakesleeana* [43].

This study aimed to find the most suitable stimulator for BA’s production from *I. obliquus*. Oleic acid and fungal elicitor were found to be more effective in promoting mycelial growth and BA’s accumulation among the tested exogenous stimulators than betulin. 1.0–2.0 g/L of oleic acid could lead to a significant increase on mycelia biomass, which was consistent with the mycelia growth of I. obliquus in previous studies [5,7]. Oleic acid was also beneficial for the accumulation of BA and 1.0 g/L was the most efficient. Additionally, Oleic acid was found to enhance *I. obliquus* triterpenoids (inotodiol, trametenolic acid, betulin and 3β-hydroxy-lanosta-8,24-dien-21-al), polyphenols and flavonoids production [5,7,44]. It appears to be the first report that the supplementation of oleic acid could promote BA production in the submerged culture of *I. obliquus*. A possible mechanism for fatty acids stimulatory effect on mycelia growth and the production of secondary metabolites (triterpenoids, polysaccharide and polyphenol) is that they were speculated to alter membrane components, enhance permeability and accelerate the uptake of nutrients from the liquid medium. However, this mechanism seemed to have been verified only on polyunsaturated fatty acids (PUFAs) [7]. Whereas this postulated mechanism hardly applied to saturated and monounsaturated fatty acids because they incorporated less well into the membranes [7,45]. Another possible mechanism is that fatty acids directly affected the synthesis of secondary metabolite [10,46]. For example, fatty acids of *Coix lacryma-jobi* oil increased polysaccharide production by directly affecting the synthesis level of phosphoglucose isomerase and α-phosphoglucomutase at different stage [10]. BA is synthesized mainly via the mevalonate pathway where acetyl-CoA is converted through a series of chemical reactions to 3-hydroxy-3-methylglutaryl-CoA (HMG-CoA), to mevalonate (MVA), to isopentenyl-pyrophosphate (IPP), to farnesyl diphosphate (FPP), to squalene, and finally to betulinic acid [7,12]. Some enzymes are involved in the synthesis of betulinic acid including HMG-CoA synthase (HMGS), HMG-CoA reductase (HMGR), farnesyl pyrophosphate synthase (FPS) and squalene synthase (SQS) [47]. Oleic acid, a kind of monounsaturated fatty acid, could influence BA production by affecting synthesis of enzyme [12]. Data of RT-qPCR confirmed that the addition of oleic acid could promote the transcriptional level of HMGR gene significantly and HMGR is the key enzyme of the mevalonate pathway. Consequently, it is likely that oleic acid increases BA’s production by up-regulating the key enzyme genes of BA synthesis. Meanwhile, oleic acid may promote the production of BA by providing acetyl-CoA, which of acetyl-CoA is the main precursor of BA. However, it is unclear if betulinic acid results only from *I. obliquus* de novo synthesis, from the transformation of birch terpenoid metabolites, or from both [48].

**Figure 7:**
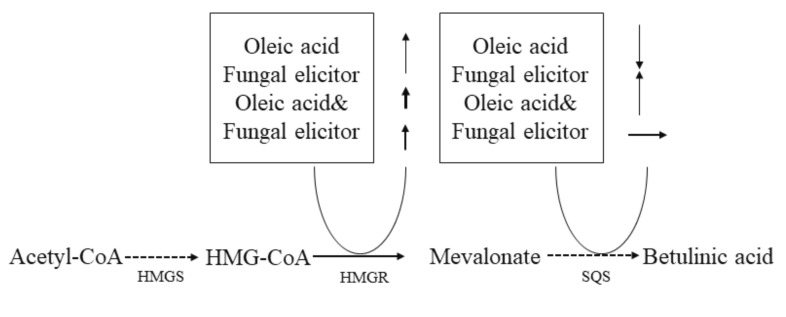
The postulated regulation way of small molecules on BA production.

For fungal elicitor derived from *A. niger*, it had a significant enhancement on the biomass and BA’s production at the concentrations of 45–60 mg/L. The results here were in partial agreement with the previous report [14]. The elicitor prepared from fungi was complicated and made up of proteins, polysacchrides, glycoproteins, peptides, oligosaccharides and lipoids [49]. The effects of different sources of fungal excitons were also different [50,51]. As early reported, fungal elicitor was able to improve the biomass, entire carotenoids yield of *Xanthophyllomyces dendrorhous* at various levels [15]. Fungal elicitors obtained from *F. oxysporum, A. niger and S. cerevisiae* had an inhibitory effect on cell growth of *G. lucidum*, while the productivity of ganoderic acid and polysaccharide was improved markedly by the stimulation of fungal elicitors [14]. In previous literatures, some researchers found that elicitors worked in plants and fungal cells by initiating a defense response in cell cultures and activating silent fungal secondary metabolism genes [52,53]. The result of RT-qPCR indicated that the existence of fungal elicitor could enhance the expression level of both HMGR and SQS. It is probable that fungal elicitor promotes the yield of BA by enhancing the expression level of key enzyme genes. As shown in Figure 7, it suggests that exogenously supplied elicitors may induce BA biosynthesis via the inducement of enzymes in *I. obliquus* through a complex system of signals.

In previous studies, BA was mainly transformed from betulin by cultured resting cells [12,13]. In the present work, oleic acid and fungal elicitor from *A. niger* were proved to be effective inducers for BA accumulation in the submerged fermentation of *I. obliquus*. And, the combination of oleic acid and fungal elicitor was more effective to acquire better growth and BA production than the individuals. This has never been mentioned in previous studies [6,7,54]. It is of great significance to set up the most appropriate transformation system by optimizing this process. To obtain high yield of BA and biocatalysts stability, it is critical to optimize the reaction parameters. In this work, the optimal amount and addition time of the two stimulators as well as fermentation period, has been determined. The optimal amount of oleic acid and fungal exciter was 1.0 g/L and 45 mg/L, respectively. The best time to add 1.0 g/L oleic acid, 45 mg/L fungal elicitor and their com-bination was the 4th day, the 4th day and the 7th day, respectively; the optimal fer-mentation time was 12,14 and 10 days, respectively, when 1.0 g/L oleic acid, 45 mg/L fungal excites and their combinations were used for induction. Thus, the addition of exogenous additives and the optimization of fermentation conditions effectively improved the yield of BA in the submerged culture of *I. obliquus*.

## 5. Conclusions

In summary, we investigated the effect of oleic acid, fungal elicitor and the combination of oleic acid and fungal elicitor on the accumulation of BA in the submerged culture of *I. obliquus*. They all could increase the production of BA and promote the growth of mycelia. The combination of oleic acid and fungal elicitor has the most significant effect. This comprehensive analysis of the production of BA and the expression levels of a few genes regulating the biosynthesis of BA that include HMGR and SQS provides the foundation to understand the working mechanism of oleic acid and fungal elicitor. The findings of this study will facilitate the further improvement of the yield of BA. Furthermore, this work can provide the basis for the development of novel and effective induction method on BA biosynthesis.

## Data Availability

The data presented in this study are available in article.
